# The Metabolic Responses to L-Glutamine of Livers from Rats with Diabetes Types 1 and 2

**DOI:** 10.1371/journal.pone.0160067

**Published:** 2016-08-04

**Authors:** Jurandir Fernando Comar, Denise Silva de Oliveira, Livia Bracht, Fumie Suzuki Kemmelmeier, Rosane Marina Peralta, Adelar Bracht

**Affiliations:** Laboratory of Liver Metabolism, University of Maringá, 87020900 Maringá, Brazil; Chi-Mei Medical Center, TAIWAN

## Abstract

There are several claims about the beneficial effects of supplementing L-glutamine to both type 1 and type 2 diabetes. The purpose of the present study was to provide detailed knowledge about the fate of this amino acid in the liver, the first organ that receives the compound when ingested orally. The study was done using the isolated perfused rat liver, an experimental system that preserves the microcirculation of the organ and that allows to measured several parameters during steady-state and pre steady-state conditions. L-Glutamine was infused in the portal vein (5 mM) and several parameters were monitored. Livers from type 1 diabetic rats showed an accelerated response to L-glutamine infusion. In consequence of this accelerated response livers from type 1 diabetic rats presented higher rates of ammonia, urea, glucose and lactate output during the first 25–30 minutes following L-glutamine infusion. As steady-state conditions approached, however, the difference between type 1 diabetes and control livers tended to disappear. Measurement of the glycogen content over a period of 100 minutes revealed that, excepting the initial phase of the L-glutamine infusion, the increased glucose output in livers from type 1 diabetic rats was mainly due to accelerated glycogenolysis. Livers from type 2 diabetic rats behaved similarly to control livers with no accelerated glucose output but with increased L-alanine production. L-Alanine is important for the pancreatic β-cells and from this point of view the oral intake of L-glutamine can be regarded as beneficial. Furthermore, the lack of increased glucose output in livers from type 2 diabetic rats is consistent with observations that even daily L-glutamine doses of 30 g do not increase the glycemic levels in well controlled type 2 diabetes patients.

## Introduction

There are several claims about the beneficial effects of supplementing L-glutamine to both type 1 and type 2 diabetes [1–5]. L-Glutamine is the physiological precursor of L-arginine for the synthesis of nitric oxide (NO·), whose production in β-cells potentiates insulin secretion. Moreover, L-glutamine combined with L-alanine constitute the main source of glutamate for the synthesis of GSH. The latter is important in helping to diminish the oxidative stress which ultimately contributes for maintaining inflammatory processes within the β-cells in diabetes [6]. L-Glutamine is also efficacious in increasing the secretion of the glucagon-like peptide-1 and when administered with a meal it reduces postprandial glycaemia in type 2 diabetic patients [7,8]. Besides improving the glucose profile L-glutamine also has a positive effect on glucose oxidation and insulin resistance [5,9]. Furthermore, in streptozotocin-induced diabetes in rats, L-glutamine supplementation improved the plasma levels of transminases, fructosamine and the lipid profile [10]. The doses of L-glutamine that are administered with a meal may reach 30 g [4]. Doses of this magnitude will necessarily lead to high L-glutamine concentrations in the portal vein, the most important entry vessel of the liver.

In the liver the main enzymes involved in L-glutamine metabolism are glutaminase (degradation) and L-glutamine syntethase (synthesis). These enzymes predominate in different regions along the hepatic acini, the former in the periportal and the latter in the perivenous zone [11,12]. The high activity of both glutaminase and L-glutamine synthetase in the liver allows to this organ to remove from and to add L-glutamine to the circulation. In consequence, the liver plays an important role in L-glutamine homeostasis [13]. Glutaminase is considered a key-enzyme for hepatic L-glutamine utilization [14]. Its activity changes under certain pathological conditions, such as diabetes, for example [15,16]. In streptozotocin-induced diabetic rats (type 1 diabetes), increases of up to 12-fold have been reported [17]. Consistently, in diabetes, the liver switches from release to net uptake of L-glutamine [18,19]. In accordance to this, an increased L-glutamine utilization was observed in hepatocytes isolated from streptozotocin-induced diabetic rats [16]. However, the experiments with isolated hepatocytes have been done by measuring either ^14^CO_2_ accumulation in the presence of L-[^14^C]glutamine [17] or glucose accumulation during 30 minutes [20]. The latter, especially, does not allow to differentiate between glycogenolysis and gluconeogenesis, an important detail if one considers that the fasting glycogen content of livers from type 1 diabetic rats is usually high [21]. Moreover, it has been shown in experiments with the isolated perfused liver from healthy rats that the utilization of L-glutamine is characterized by a pronounced lag phase so that usually much more than 30 minutes are required for attaining steady-state conditions [22]. In this way, single measurements of L-glutamine metabolism in isolated hepatocytes after 30 minutes incubation could be revealing an untrue picture of the real differences between healthy and diabetic rats if the time dependence is also changed, as it has been demonstrated to occur in rats bearing the Walker256 tumor [22].

From what was exposed above it can be deduced that the claims about the beneficial effects of supplementing L-glutamine to both type 1 and type 2 diabetic patients [1–5,8] need to be complemented by a more detailed knowledge about the fate of this amino acid in the liver, the first organ that receives the compound when ingested orally. To produce this knowledge was precisely the purpose of the present work in which livers from rats with types 1 and 2 diabetes were perfused with L-glutamine until steady-state conditions were attained. Several parameters, such as gluconeogenesis, ureagenesis and glycogen contents were measured. In the interpretation of the results, consideration was given to the glycogen content of the liver from diabetic rats [21] as well as to the delayed kinetics of the hepatic response to L-glutamine [22].

## Material and Methods

### Materials

The liver perfusion apparatus was built in the workshops of the University of Maringá. All enzymes and coenzymes used in the enzymatic assays were purchased from Sigma Chemical Co. (St Louis, USA). All standard chemicals were from the best available grade (98–99.8% purity).

### Animals and diabetes induction

Male Wistar rats (*Rattus novergicus*) weighing 220–250 g and fed with a standard laboratory diet (Nuvilab^®^) were used in all perfusion experiments. Type 1 diabetes was induced by injecting adult rats with streptozotocin [21]. Streptozotocin was dissolved in citrate buffer (10 mM; pH 4.5) and a single dose of 50 mg/kg was injected intraperitoneally. After 12 days blood glucose of fed rats was measured and animals presenting glycemic levels equal or above 16 mM were selected for the experiments. Control rats were injected intraperitoneally with citrate buffer (10 mM, pH 4.5).

Neonatal type 2 diabetes mellitus was induced as described previously [23,24]. Male newborn (2 days old) Wistar rats were injected intraperitoneally with streptozotocin (150 mg/kg) dissolved in citrate buffer (10 mM; pH 4.5). Seven weeks later, diabetes was confirmed by urine glucose levels, 24 hours urinary volume, and water intake.

All experiments were done in accordance with the internationally accepted recommendations in the care and use of animals. The protocols were approved by the Ethics Committee of Animal Experimentation of the University of Maringá (Protocol 098/10-CEEA). Surgery was performed under sodium pentobarbital anesthesia, and all efforts were made to minimize suffering. The criterion of anesthesia was the lack of body or limb movement in response to a standardized tail clamping stimulus.

### Liver perfusion

For the surgical procedure, the rats were anesthetized by intraperitoneal injection of sodium pentobarbital (50 mg/kg). Hemoglobin-free, non-recirculating perfusion was done [25]. After cannulation of the portal and cava veins the liver was positioned in a plexiglass chamber. The constant flow was provided by a peristaltic pump (Minipuls 3, Gilson, France) and was adjusted between 30 and 35 mL min^-1^, depending on the liver weight. The perfusion fluid was Krebs/Henseleit-bicarbonate buffer (pH 7.4), saturated with a mixture of oxygen and carbon dioxide (95:5) by means of a membrane oxygenator with simultaneous temperature adjustment at 37°C. The composition of the Krebs/Henseleit-bicarbonate buffer is the following: 115 mM NaCl, 25 mM NaHCO_3_, 5.8 mM KCl, 1.2 mM Na_2_SO_4_, 1.18 mM MgCl_2_, 1.2 mM NaH_2_PO_4_ and 2.5 mM CaCl_2_.

### Analytical

Samples of the effluent perfusion fluid were collected according to the experimental protocol and analyzed for their metabolite contents. The following compounds were assayed by means of standard enzymatic procedures: glucose [26], urea [27], ammonia [28], lactate [29], alanine [30] and glutamate [31]. The oxygen concentration in the outflowing perfusate was monitored continuously, employing a teflon-shielded platinum electrode adequately positioned in a plexiglass chamber at the exit of the perfusate [25,32]. Metabolic rates were calculated from input-output differences and the total flow rates and were referred to the wet weight of the liver.

Glycogen was determined in isolated perfused livers. Portions of approximately 2 g were freeze-clamped with liquid nitrogen. This sample was homogenized and extracted with 8 mL of 6% HClO_4_. The supernatant was neutralized with K_2_CO_3_ and used for enzymatic glycogen assay [33].

### Treatment of data

The metabolic rates were expressed as μmol per minute per gram liver wet weight (μmol min^-1^ g^-1^). Statistical analysis of the data was done by means of the Statistica^TM^ program (Statsoft^®^, Tulsa, USA). Monovariate variance analysis or two-way variance analysis with post-hoc testing were applied according to the context; p ≤ 0.05 was adopted as the criterion of statistical significance. Numerical interpolation for determining the times of half-maximal responses to L-glutamine infusion (t_½_) was done by means of the Scientist^®^ Program from MicroMath Scientific Software (Salt Lake City, USA).

## Results

### Glucose and lactate output and oxygen uptake

**Fig 1:**
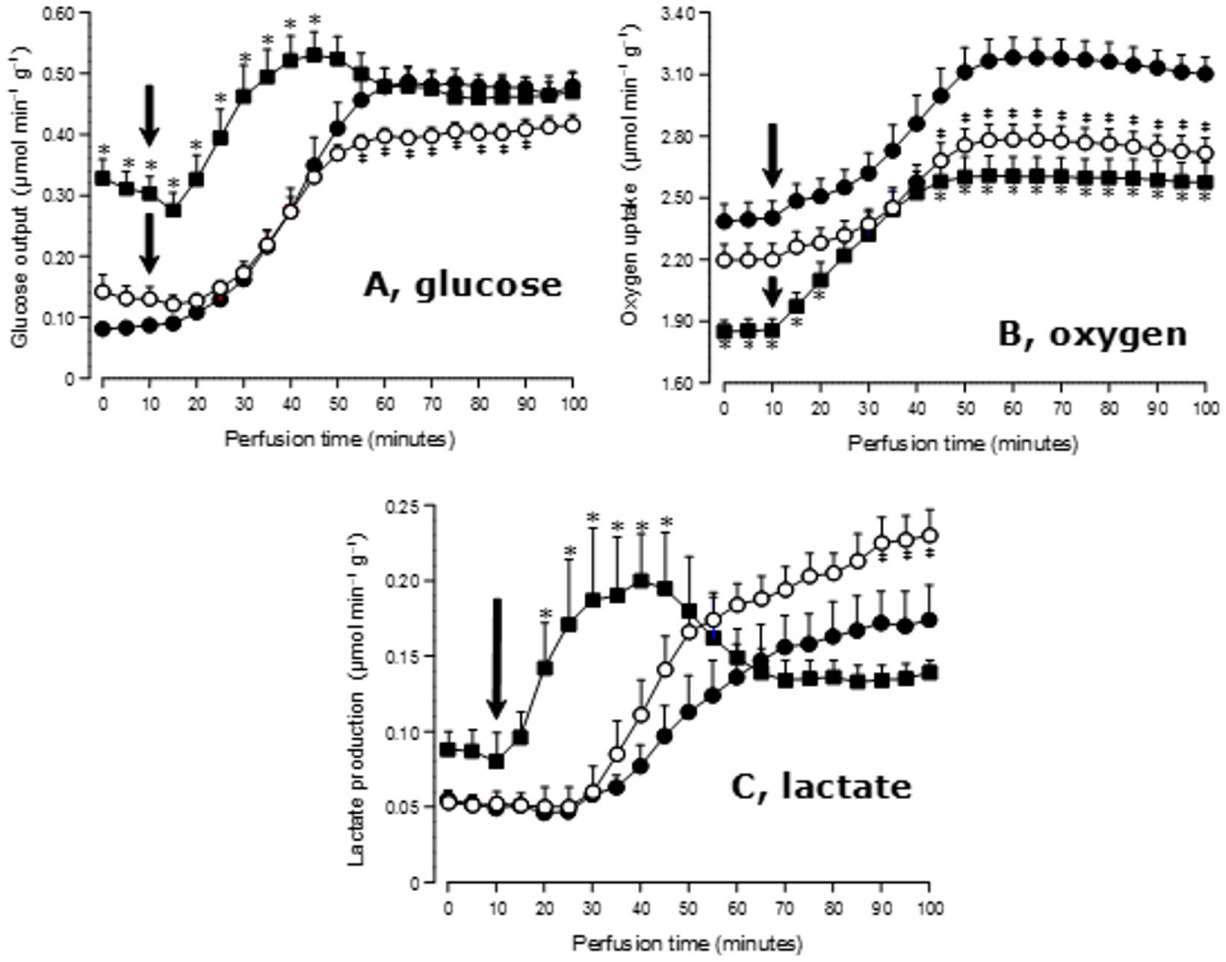
Time courses of glucose and lactate output and oxygen uptake during L-glutamine infusion (5 mM) in livers from control and diabetic rats after a 24 hours fasting period. L-Glutamine infusion was initiated at 10 minutes perfusion time, as indicated by the black arrows. Legends: control, ●—●; type 1 diabetes, ■—■; type 2 diabetes, ○—○. Statistically significant differences between control and type 1 diabetes or control and type 2 diabetes are indicated by the symbols * and **‡**, respectively.

The metabolism of L-glutamine was investigated in perfused livers from 24-hours fasted rats in order to minimize interference by endogenous glycogen. After a short pre-perfusion time for allowing oxygen uptake stabilization the infusion of 5 mM L-glutamine was initiated. This should mimic the sudden entry of a glutamine load into the portal vein in consequence of the ingestion of a great dose of this amino acid. Besides oxygen uptake, the output of glucose, lactate, ammonia, urea, alanine and glutamate were monitored. The time courses of the changes in glucose and lactate output and the changes in oxygen consumption are shown in Fig 1. The first two parameters reflect the carbon fluxes derived from L-glutamine after removal of the amine group (nitrogen).

Fig 1A shows that the basal rate of glucose output in livers from type 1 diabetic rats was more than 3-fold higher than in those from control and type 2 diabetic rats. The last two were equal. The introduction of L-glutamine increased glucose production as expected, but the response of livers from type 1 diabetic rats was much faster. Both livers from control and type 2 diabetic rats presented the characteristic response delays already reported in a previous work [22]. A quantitative evaluation of this delay is provided by the time for half-maximal increase (t_½_), which was evaluated by numerical interpolation. Mean values of t_½_ are listed in Table 1. For type 1 diabetes the t_½_ was approximately 40% of that one computed for the controls. By virtue of their faster response, the livers from type 1 diabetic rats presented considerably higher rates of glucose output during the first 30 minutes of L-glutamine infusion. At 30 minutes perfusion time, for example, glucose output in livers from type 1 diabetic rats was 2.7-fold higher. After 40 minutes perfusion time, however, when steady-state conditions had apparently been established, the rates of glucose output in both livers from control and from type 1 diabetic rats became equal. Livers from type 2 diabetic rats, on the other hand, tended to present somewhat diminished rates of glucose output when steady-state conditions were reached.

**Table 1:** Half-maximal rate times (t_½_) of L-glutamine action on several metabolic fluxes.

Metabolic fluxes		t_½_ (minutes)	
	Control	Type 1 diabetes	Type 2 diabetes
**Glucose production**	32.68±1.89 (n = 11)	19.83±1.53* (n = 12)	29.99±1.30 (n = 12)
**Ammonia production**	26.91±2.45 (n = 6)	14.12±2.42* (n = 7)	23.47±0.78 (n = 6)
**Urea production**	31.52±3.02 (n = 6)	16.16±3.69* (n = 6)	23.80±0.95* (n = 6)
**Lactate production**	43.23±3.94 (n = 6)	16.65±3.74* (n = 6)	34.90±2.23 (n = 5)
**Oxygen uptake**	28.95±1.82(n = 11)	19.01±1.55* (n = 12)	27.38±1.46 (n = 12)

**Fig 2:**
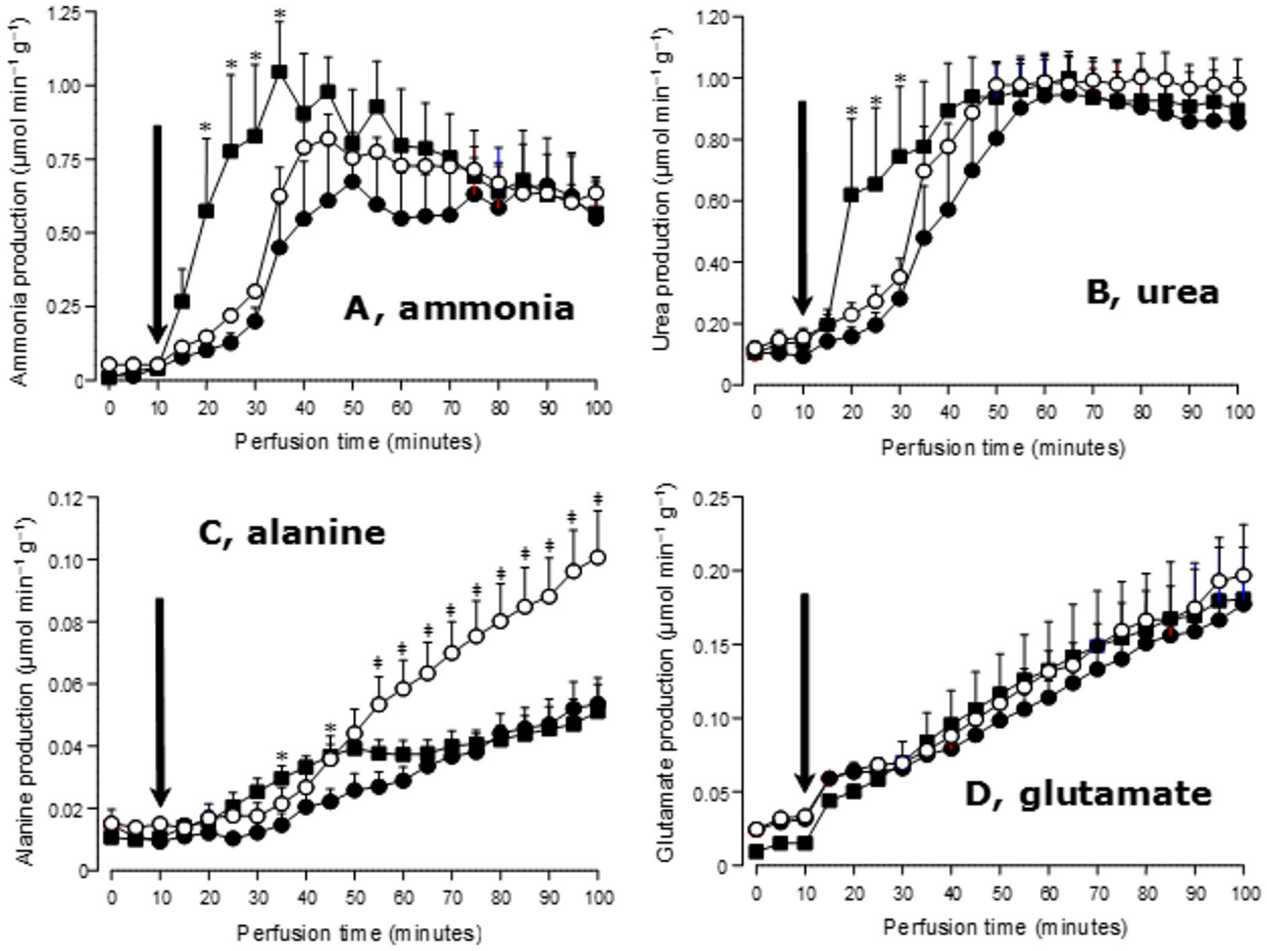
Time courses of ammonia, urea, alanine and L-glutamate output during L-glutamine infusion (5 mM) in livers from control and diabetic rats after a 24 hours fasting period. L-Glutamine infusion was initiated at 10 minutes perfusion time, as indicated by the black arrows. Control, ●—●; type 1 diabetes, ■—■; type 2 diabetes, ○—○. Statistically significant differences between control and type 1 diabetes or control and type 2 diabetes are indicated by the symbols * and **‡**, respectively.

The t_½_ values are expressed in terms of time after initiation of L-glutamine infusion and were obtained by numerical interpolation (Stineman's interpolation) within each individual glutamine response curve of the experimental series illustrated by Figs 1 and 2. Data are means ± SEM and asterisks are used to indicate values differing statistically from the corresponding controls according to the Student-Newman-Keuls test applied after monovariate variance analysis (*p* ≤ 0.05).

Oxygen uptake of livers from type 1 diabetic rats was smaller before and after L-glutamine infusion, as revealed by Fig 1B. In the case of livers from type 2 diabetic rats oxygen uptake was smaller after stimulation by L-glutamine. The response to L-glutamine of livers from type 1 diabetic rats was also faster, as revealed by the t_½_ value in Table 1.

Lactate output is shown in Fig 1C. Basal rates tended to be higher in livers from type 1 diabetic rats, but without statistical significance at the 5% level. The response of livers from type 1 diabetic rats was much faster than that of livers from control and type 2 diabetic rats (see t_½_ values in Table 1). As it happened with glucose output, during the first 30 minutes lactate output in livers from type 1 diabetic rats was considerably higher than in livers from control and type 2 diabetic rats. After 40 minutes perfusion time the lactate output in livers from control and type 1 diabetic rats declined progressively to steady-state levels similar to those of control livers. The opposite occurred in livers from type 2 diabetic rats in which the steady-state rates of lactate output tended to become higher than those of livers from control and type 1 diabetic rats.

### Nitrogen fluxes

The kinetics of the output of ammonia, urea, alanine and glutamate during L-glutamine infusion is illustrated by Fig 2. The basal rates, i.e., before the onset of L-glutamine infusion, were minimal for the four parameters, irrespective of the condition. Ammonia production (Fig 2A) increased upon L-glutamine infusion. The response was faster in livers from type 1 diabetic rats when compared to the livers from control and type 2 diabetic rats (see Table 1 for the t_½_ values). Ammonia release was considerably higher in livers from type 1 diabetic rats during the first 20 minutes of the infusion; after 30 minutes perfusion time, however, all differences between the three groups vanished. Urea production showed a similar pattern (Fig 2B): a rapid response in livers from type 1 diabetic rats and slow responses in livers from control and type 2 diabetic rats, followed by a triple convergence toward similar steady-states. In consequence, urea production was considerably higher in livers from type 1 diabetic rats during the first 15 minutes of the L-glutamine infusion.

Alanine production, which is shown in Fig 2C, tended to be higher in livers from type 1 diabetic rats when compared to the controls during the first minutes after the onset of the infusion, but the rates were small. More remarkable is the great increase experienced in livers from type 2 diabetic rats after 40 minutes of L-glutamine infusion (50 minutes perfusion time). This increase proceeded linearly with time until the end of the perfusion experiment (100 minutes). At this time the rate of alanine release in livers from type 2 diabetic rats was nearly 2-fold higher than that in livers from control or type 1 diabetic rats.

Glutamate production, shown in Fig 2D, was nearly identical for the three groups. Remarkably, it increased almost linearly with time from the 10^th^ to the 100^th^ minute.

### Glucose output in livers from type 1 diabetic rats and glycogen content

**Fig 3:**
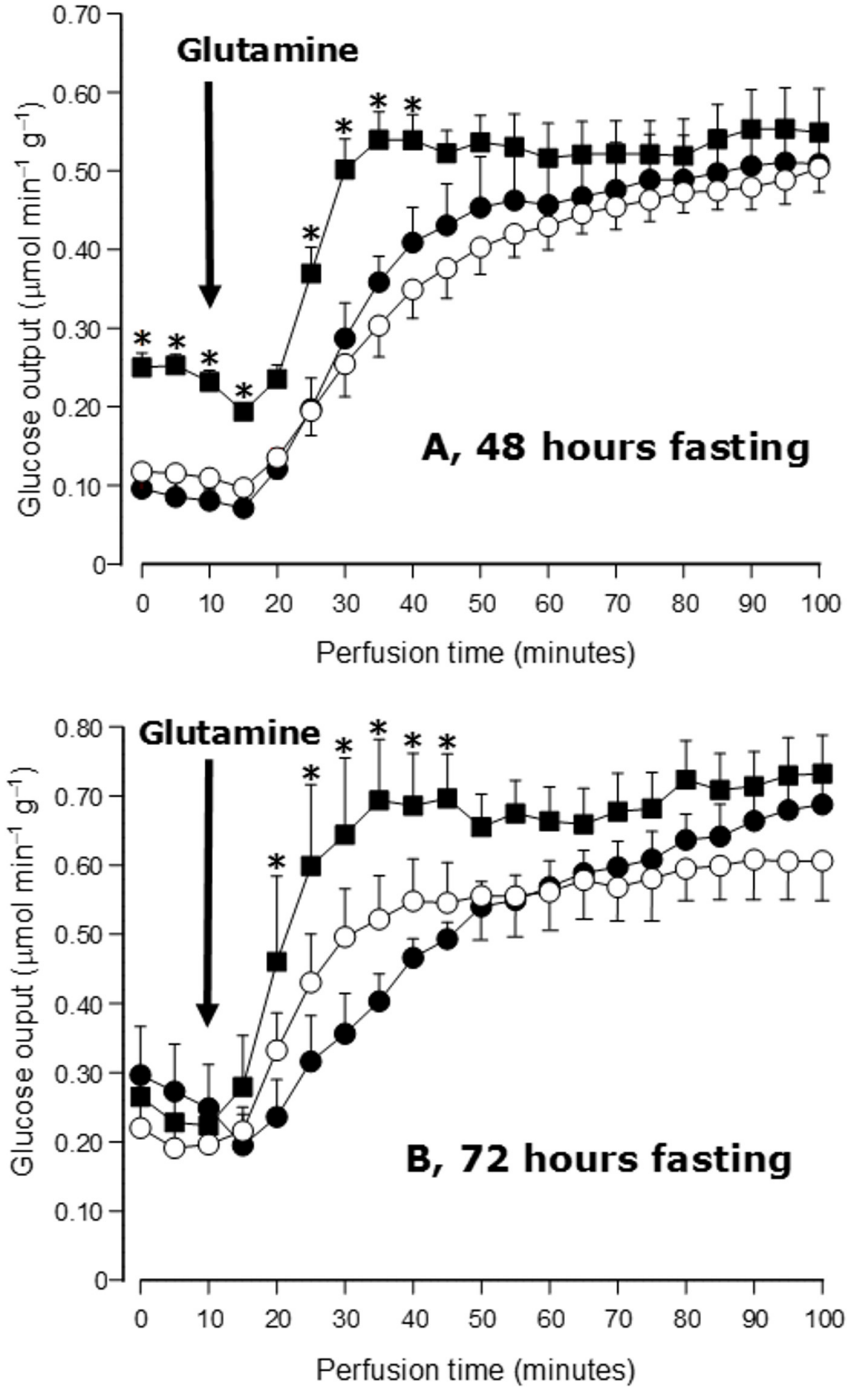
Time courses of glucose output during L-glutamine infusion (5 mM) in livers from control and diabetic rats after fasting periods of 48 and 72 hours. L-Glutamine infusion was initiated at 10 minutes perfusion time, as indicated by the black arrows. Legends: control, ●—●; type 1 diabetes, ■—■; type 2 diabetes, ○—○. Statistically significant differences between control and type 1 diabetes are indicated by the symbol *.

It is known that rats with type 1 diabetes maintain relatively high levels of hepatic glycogen even after a 24 hours fast [21,34], a phenomenon that also causes high basal rates of glucose output (see Fig 1A). In an attempt of reducing these basal rates, the fasting periods were increased to 48- and 72-hours and glucose production was measured [34]. Fig 3 shows the results that were obtained. After a 48-hours fast (Fig 3A) the basal rate of glucose output in livers from type 1 diabetic rats was still higher in comparison with the livers from control or type 2 diabetic rats. Introduction of L-glutamine produced a rapid increase which stabilized at levels similar to those of control livers. The response of livers from type 2 diabetic rats was similar to the response of the control livers. After a 72-hours fast (Fig 3B) the basal rates of glucose output were pratically the same in livers from control and diabetic animals. Here again the initial rapid increase of glucose production in livers from type 1 diabetic rats was present and the response was stable. Livers from type 2 diabetic rats also tended to respond more rapidly, statistical significance, however, was lacking.

### Glycogenolysis, glycogenesis and gluconeogenesis during L-glutamine infusion

**Fig 4:**
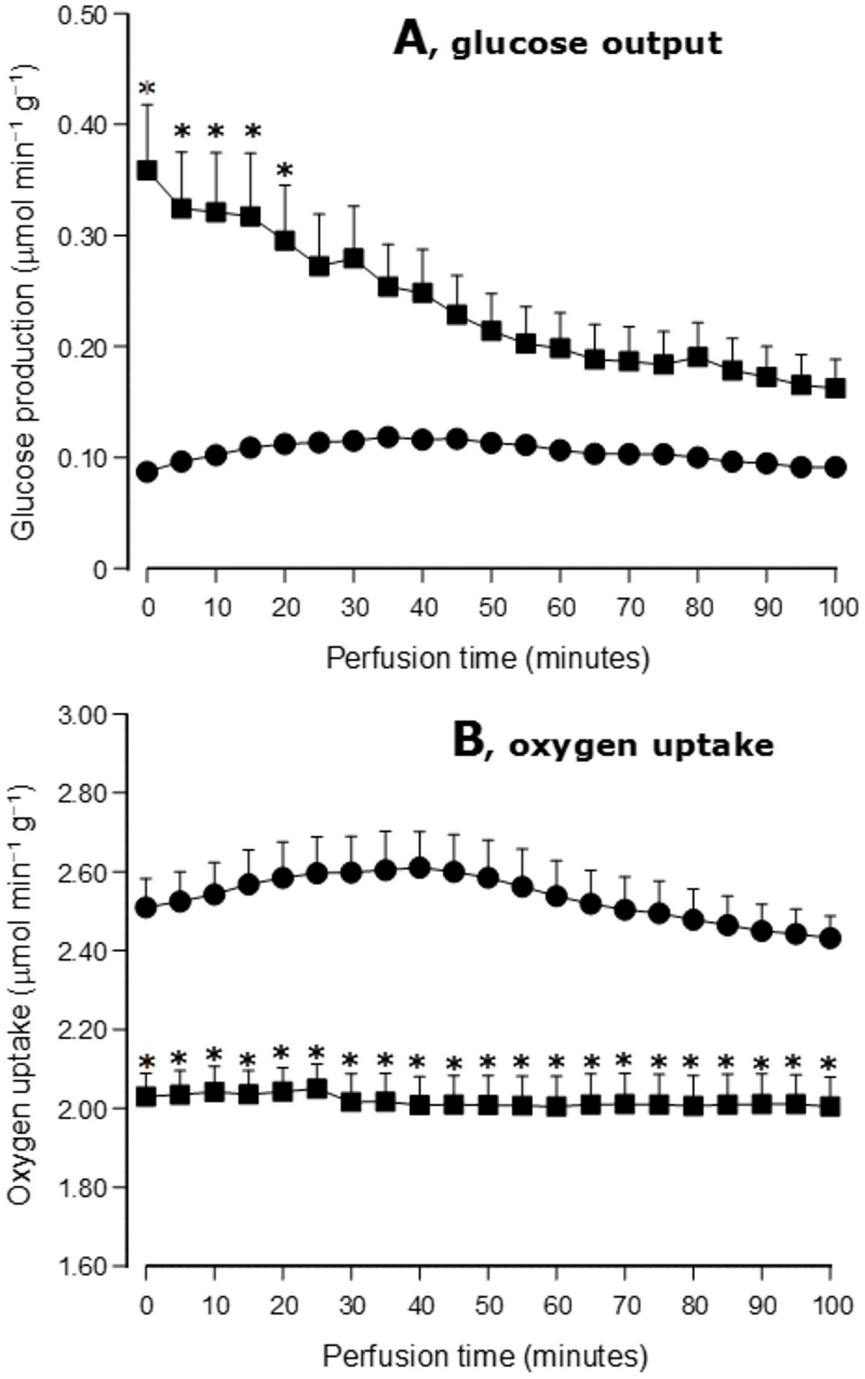
Time courses of glucose output and oxygen uptake in livers from 24 hours fasted rats under substrate-free perfusion. Legends: control, ●—●; type 1 diabetes, ■—■. Statistically significant differences between control and type 1 diabetes are indicated by the symbols *.

The high glycogen levels of livers from type 1 diabetic rats and the kinetics of glucose output following L-glutamine infusion lead to the question about the relative contribution of glycogenolysis and gluconeogenesis. In order to answer this question the glycogen levels of livers from type 1 diabetic rats and control rats (24-hours fasted) were measured at 10 and 100 minutes perfusion time, i.e., before and at the end of L-glutamine infusion. These levels were compared with the total amounts of glucose that were produced from 10 to 100 minutes perfusion time. Control experiments were also done in which glucose release and oxygen uptake were measured without substrate infusion. The results shown in Fig 4 reveal low, but steady rates of glucose output in control livers during the whole perfusion time and higher, but declining rates in livers from type 1 diabetic rats. Oxygen uptake, which was higher in control livers, showed almost no changes during the whole perfusion time. Table 2 shows the measured glycogen contents after the pre-perfusion period (arbitrarily set as 10 minutes perfusion time in Figs 1 and 4) and the total glucose output (Δglucose output) during substrate-free perfusion and perfusion in the presence of L-glutamine. The latter was evaluated as the area under the curves between 10 and 100 minutes perfusion time. Derived parameters are gluconeogenesis and the differences in glycogen content (Δglycogen content) between 10 and 100 minutes, the negative sign meaning net glycogenolysis and the positive sign net glycogenesis. Gluconeogenesis was estimated as the difference between the change in glycogen content and the total glucose output. Control livers presented very low glycogen levels after the pre-perfusion period which includes the time between anesthesia and surgical preparation of the liver and stabilization of oxygen uptake. In livers from type 1 diabetic rats the glycogen levels were much higher than in the control livers, but substantially lower than those found in vivo [34]. This is probably due to the high rates of glucose output during the preperfusion period. In absolute and relative terms net glycogen degradation was small during the 100 minutes substrate-free perfusion in control livers (-22.7%), but high in livers from type 1 diabetic rats (-81.3%). In control livers, gluconeogenesis during substrate-free perfusion was relatively high. In livers from type 1 diabetic rats gluconeogenesis was negligible, the total glycogenolysis pratically accounting for the total glucose output during the whole substrate-free perfusion period. The infusion of L-glutamine produced some net glycogen synthesis (+63.2% above the 10 minutes levels) in control livers and a reduction in net glycogenolysis in livers from type 1 diabetic rats. The total glucose output during L-glutamine infusion was higher in livers from type 1 diabetic rats (+28.5%), but this difference was mainly due to glycogenolysis, because gluconeogenesis was similar for both healthy and diabetic condition.

**Table 2:** Glycogen contents, total glucose output (Δglucose output), changes in glycogen contents (Δglycogen content) and gluconeogenesis of livers from 24-hours fasted rats during substrate-free perfusion and perfusion with 5 mM L-glutamine.

Condition of the perfusion fluid	Perfusion time (minutes)	Control				Type 1 diabetes			
		Glycogen content	ΔGlucose output	ΔGlycogen content	Gluconeo-genesis	Glycogen content	ΔGlucose output	ΔGlycogen content	Gluconeo-genesis
*μmol glucosyl units per gram liver wet weight*									
Pre-perfusion	10	1.58±0.08 (n = 6)^a^^,^^α^^,^^β^	—	—	—	23.44±4.29 (n = 4)^a^^,^^γ^^,^^δ^	—	—	—
Substrate-free	100	1.22±0.02 (n = 3)^α^	9.60±0.31 (n = 3)^c^	-0.36	9.24	4.36±1.27 (n = 11)^γ^	19.14±2.29 (n = 10)^c^	-19.08	0.06
L-Glutamine (5 mM)	100	2.58±0.33 (n = 4)^b^^,^ ^β^	31.68±2.02 (n = 11)^d^	+1.00	32.68	11.96±0.96 (n = 12)^b^^,^^δ^	40.73±2.88 (n = 12)^d^	-11.48	29.25

^**a,b**^*p* < 0.001;

^**c**^*p* = 0.049;

^**d**^*p* = 0.042;

^α^*p* = 0.018;

^β^*p* = 0.007;

^γ^*p* < 0.001;

^δ^*p* = 0.001.

The experiments were done according to the protocols illustrated by Fig 1 (glutamine infusion) and Fig 4 (substrate-free perfusion). For glycogen determination the livers were freeze-clamped in liquid nitrogen at 10 or 100 minutes perfusion time. Total glucose output (Δglucose output) was computed as the area under the corresponding curves in Figs 1A and 4A. Data are means ± SEM and the superscripts of each pair of values refer to the various *p* values given in the bottom (Student's t test); *p* values > 0.05 where omitted. Latin letters were used for comparing columns and Greek letters when comparing lines.

## Discussion

Type 1 diabetes induced modifications in the hepatic metabolism of L-glutamine. These modifications, however, were mainly transient in nature. Type 1 diabetes only accelerated the initial response to the introduction of L-glutamine, the subsequent steady-state rates, however, were similar to those in livers from control rats. In the case of lactate and ammonia production the phenomenon included even a downregulation after 20 or 30 minutes. These observations suggest that the velocity of hepatic transformation of L-glutamine in vivo, if steady-state conditions predominate, is probably similar in both healthy (control) and type 1 diabetic rats. An accelerated transformation of L-glutamine in rats with type 1 diabetes would thus be restricted to episodes of transition between two different steady-states. It is appropriate to emphasize that only type 1 diabetes promotes these alterations in L-glutamine metabolism. Type 2 diabetes was pratically without action on the hepatic L-glutamine metabolism with the important exception of L-alanine production which was increased (see below). All these conclusions are opposed to the notions that were drawn from measurements of L-glutamine metabolism in isolated hepatocytes in which the accumulation of metabolites was measured after 30 minutes, i.e., without following the response over the entire incubation time [17,20].

Concerning the shorter half-maximal times for L-glutamine metabolism, the action of type 1 diabetes was similar to that found in rats bearing the Walker-256 tumor [22]. Differences in the rate of transport of L-glutamine into the hepatocytes are not a likely cause for the phenomenon, because no significant changes in Na^+^-dependent and Na^+^-independent L-glutamine transport across rat liver sinusoidal membranes have been found [35]. If the changes in the half-maximal times are not caused by L-glutamine transport into the hepatocyte, their causes could be linked to the enzyme glutaminase which catalyzes the initial and irreversible step of L-glutamine metabolism. The flux control coefficient of glutaminase, a measure of how intensely the enzyme controls the whole pathway, has been reported to be equal to 0.96 whereas values of 0.51 and -0.46 have been attributed, respectively, to the cell influx and efflux systems [14]. These values must be regarded with some caution because they have been evaluated after 10 minutes incubation periods of labeled L-glutamine with isolated hepatocytes and in the perfused liver of normal rats at least the L-glutamine metabolism is small during the first 10 minutes. The observation that type 1 diabetes does not affect hepatic L-glutamine transport [35], however, is a strong indication that glutaminase exerts a key-role in L-glutamine transformation. In this respect it must be emphasized that this enzyme is subject to complex cellular control and that knowledge of many of its regulatory factors and properties is still incomplete. The enzyme can be stimulated by glucagon and by interaction with the mitochondrial membrane, for example [36,37]. This long-term stimulation persists even after isolation of mitochondria [36], so that it probably also persists during liver perfusion. The high levels of glucagon in type 1 diabetes [38,39] and the low levels of insulin [40] are probably an important cause for the elevated levels of glutaminase in this disease [15]. This set of observations, which reflect the long-term regulation of glutaminase, most likely explains the initial higher rates of L-glutamine metabolism that were found in the type 1 diabetes condition in as much as no such phenomenon was found in the livers of rats with type 2 diabetes, which do not normally present higher glucagon levels [41]. It is also evident, however, that during the course of L-glutamine infusion divergent events must have taken place in the livers of healthy, type 2 and type 1 diabetic rats. The first two presented a sigmoidal time dependence with an accentuated increment in L-glutamine metabolism during the period of 20 to 40 minutes L-glutamine infusion, suggesting activation of glutaminase by means of a glucagon-independent mechanism. In the livers from type 1 diabetic rats, where the initial rate of metabolism was already high, no further stimulation took place. Actually a tendency toward diminution was even apparent, suggesting also a decrease in the glutaminase activity. The overall conclusion is, thus, that the response versus time curves obtained for most parameters with livers from healthy, type 2 and type 1 diabetic rats tended to converge toward equality at longer perfusion times. This phenomenon most likely results from the interplay of one or more short-term regulatory effects. It is impossible to divise with certainty the events underlying these responses. However, it is known that metabolites such as ammonia, bicarbonate, cysteine, leucine and ADP and pH changes can work as short-term regulators of the glutaminase activity [40,42,43]. The exact interplay of these and other factors is still not well understood. One cannot exclude, however, that their concentrations may have changed differently with time in livers from healthy and diabetic rats, leading to different responses.

The contribution of glycogenolysis to glucose output in livers from fasted control rats was minimal, as revealed by the glycogen levels at various times in the presence and absence of L-glutamine. The small basal rates of glucose production in these livers were, thus, the result of gluconeogenesis from endogenous substrates. These substrates are most likely glycogenic amino acids. At least 12 aminoacids are released by substrate-free perfused livers at a total rate of 0.5 μmol min^-1^ g^-1^ [44]. This rate of amino acid release is compatible with the basal glucose production rate of up to 0.1 μmol min^-1^ g^-1^ that is normally found in livers from 24 hours fasted control rats [45,46]. Higher concentrations of amino acids can even conduct to net glycogen accumulation, as it was the case when L-glutamine was introduced. This is in contrast with the net glycogenolytic activity that was found in both the absence and presence of L-glutamine in livers from 24-hours fasted type 1 diabetic rats, a phenomenon that is consistent with their high glycogen levels [34]. Although glycogenolysis and gluconeogenesis were both contributing to the net glucose release in livers from type 1 diabetic rats, the initial burst that followed L-glutamine infusion was most likely due to gluconeogenesis. This is corroborated by similar bursts in other parameters strictly dependent on L-glutamine transformation (urea and lactate production, for example) and also by the fact that the same burst was also present in livers from 48- and 72-fasted type 1 diabetic rats whose basal rates of glucose release were very close to those found in the control condition. Consistently, at these fasting times the hepatic glycogen levels of control, type 1 diabetic and type 2 diabetic rats were pratically the same [21].

With respect to the gluconeogenic capacity of hepatocytes from diabetic rats, contradictory observations are found in the specialized literature. Experiments with isolated hepatocytes of alloxan diabetic rats have lead to the conclusion that hepatic gluconeogenesis from alanine, pyruvate and fructose is considerably increased in this preparation [47]. Experiments with the isolated perfused rat liver, however, revealed that gluconeogenesis from lactate, glycerol and sorbitol is not affected by alloxan- and streptozotocin-induced diabetes (type 1 diabetes), whereas gluconeogenesis from alanine is absent in these animals [47]. On the other hand, there is also a report of diminished rates of gluconeogenesis from lactate in perfused livers from alloxan diabetic rats [45]. Similar results were obtained in the perfused liver of type 2 diabetic rats where gluconeogenesis from lactate, alanine and glycerol was clearly diminished [24]. The high basal rates of glycogenolysis due to the high glycogen levels and the way by which the gluconeogenic activity was calculated from the total rates of glucose release could have contributed to these different reports. It is quite apparent from the results of the present investigation that measurement of gluconeogenesis in livers from type 1 diabetic rats must take into account the relatively high glycogen levels which are unavoidably associated with high and declining rates of glucose output. Gluconeogenesis can be evaluated by using radioactive precursors which allow to measure newly formed glucose in a specific manner or, alternatively, as it has been done in the present work, by simultaneous measurements of the glycogen levels so that the appropriate corrections can be done. Simple subtraction of the very high basal rates after infusion of a gluconeogenic substrate [48,49] is a risky procedure, likely to lead to erroneous evaluations of the true gluconeogenic activity. In the case of L-glutamine neither type 1 diabetes nor type 2 diabetes seem to conduct to a stable higher hepatic gluconeogenic capacity. If this phenomenon occurs *in vivo* it depends on circulating factors that are absent in the isolated perfused liver.

The basal rates of oxygen uptake of livers from type 1 diabetic rats were lower than those of livers from control and type 2 diabetic rats. This confirms previous reports [21]. The infusion of L-glutamine did not eliminate this difference. Although different rates of oxygen consumption must have a metabolic meaning, it is difficult to define it with precision. In a previous study it has been shown that type 1 diabetic rats presented almost normal ATP levels, but lower ADP levels [21]. It seems, thus, that oxygen uptake in livers from type 1 diabetic rats is fairly well adjusted to their energy requirements.

The hepatic L-glutamate production from L-glutamine was not increased by type 1 or type 2 diabetes. This observation speaks against an enhanced contribution of the liver as a source of L-glutamate for the pancreatic GSH synthesis in the diabetic condition [6]. The L-alanine output in livers from type 2 diabetic rats, however, increased steadily during the whole infusion period of 90 minutes. This finding is similar to the observation of an increased L-alanine production from labeled L-glutamine in non-insulin dependent diabetic subjects (type 2 diabetes) [50]. It should also be remarked that there are several reports of elevated levels of L-alanine in the blood of type 2 diabetic subjects [51,52]. It is, thus, possible that hepatic transformation of L-glutamine into L-alanine is an important source of the elevated levels of L-alanine normally present in the plasma of type 2 diabetic subjects. It is plausible that this may happen after a L-glutamine load of 30 g in diabetic patients. L-Alanine is important for the pancreatic β-cells where it is consumed at high rates (with increased ATP production) and where it exerts insulinothropic effects [53,54]. From the viewpoint of the hepatic L-alanine production, thus, the oral intake of L-glutamine can be regarded as an additional health benefit in the case of type 2 diabetes, which also includes increased glucose uptake and diminished insulin resistance [5,9]. The main characteristics of type 2 diabetes in humans were also detected in the model (neonatal streptozotocin induced diabetic rat) used in the present study, as for example mild fasting hyperglycemy, high insulin levels and some degree of insulin resistance [55,56,57]. However, the behavior of livers from type 2 diabetic rats with respect to L-glutamine metabolism was essentially that one observed with livers from healthy rats if one excepts the already mentioned increased L-alanine production. The fasting hepatic glycogen content of livers from type 2 diabetic rats is also within the normal range [21], in sharp contrast to what was found in livers from type 1 diabetic rats. It seems, thus, that hepatic insulin resistance and all other phenomena induced by type 2 diabetes, have a relatively mild influence on the metabolic fate of L-glutamine.

## Conclusion

The results allow to conclude that a sudden load of L-glutamine in the portal vein can generate a temporarily increased glucose output in type 1 diabetes, which is probably due to enhanced gluconeogenesis during a period of 30 minutes. This enhanced glucose output may be accompanied by an equally enhanced urea and ammonia production. After this time the hepatic glucose output in type 1 diabetes equalled that found in healthy rats. The same was true for all other indicators of L-glutamine metabolism. This temporarily enhanced hepatic glucose output in type 1 diabetes did not occur in type 2 diabetes. In the latter, however, L-alanine production was substantially increased, suggesting that the liver might be the main responsible for the increased L-alanine levels in the plasma during type 2 diabetes. The lack of increased glucose output in livers from type 2 diabetic rats is consistent with observations that even daily L-glutamine doses of 30 g do not increase the glycemic levels in well controlled type 2 diabetes patients.
